# A De Novo Transcriptomics Approach Reveals Genes Involved in Thrips Tabaci Resistance to Spinosad

**DOI:** 10.3390/insects12010067

**Published:** 2021-01-13

**Authors:** Ran Rosen, Galina Lebedev, Svetlana Kontsedalov, David Ben-Yakir, Murad Ghanim

**Affiliations:** Department of Entomology, Volcani Center, Rishon LeZion 7505101, Israel; ranros83@gmail.com (R.R.); galinal@volcani.agri.gov.il (G.L.); nasvetla@yahoo.com (S.K.); benyak@volcani.agri.gov.il (D.B.-Y.)

**Keywords:** *Thrips tabaci*, transcriptome, spinosad, resistance, fitness cost

## Abstract

**Simple Summary:**

Insect pest resistance to pesticides is a major problem that limits efficient management and thus decreases productivity for farmers and increases the use of harmful materials that contaminate the environment and endanger humans and beneficial organisms. A major approach for resistance management is understanding how insect pest field populations develop resistance at biological and molecular levels. In this manuscript, we studied the molecular and biological basis of resistance among tobacco thrips “onion thrips” resistant the susceptible populations to spinosad, a major insecticide that has been extensively used in recent years, by using *de novo* transcriptomics and bioassays. We found that resistance to spinosad can be metabolic by increasing the levels of detoxifying enzymes among the resistant population; however, resistant populations are more fecund compared to susceptible one, suggesting the lack of fitness cost of the resistance trait.

**Abstract:**

The onion thrip, *Thrips tabaci* (Thysanoptera: Thripidae) is a major polyphagous pest that attacks a wide range of economically important crops, especially Allium species. The thrip’s damage can result in yield loss of up to 60% in onions (*Allium cepa*). In the past few decades, thrip resistance to insecticides with various modes of actions have been documented. These include resistance to spinosad, a major active compound used against thrips, which was reported from Israel. Little is known about the molecular mechanisms underlying spinosad resistance in *T. tabaci*. We attempted to characterize the mechanisms involved in resistance to spinosad using quantitative transcriptomics. Susceptible (LC50 = 0.6 ppm) and resistant (LC50 = 23,258 ppm) thrip populations were collected from Israel. An additional resistant population (LC50 = 117 ppm) was selected in the laboratory from the susceptible population. De novo transcriptome analysis on the resistant and susceptible population was conducted to identify differently expressed genes (DGEs) that might be involved in the resistance against spinosad. In this analysis, 25,552 unigenes were sequenced, assembled, and functionally annotated, and more than 1500 DGEs were identified. The expression levels of candidate genes, which included cytochrome P450 and vittelogenin, were validated using quantitative RT-PCR. The cytochrome P450 expression gradually increased with the increase of the resistance. Higher expression levels of vitellogenin in the resistant populations were correlated with higher fecundity, suggesting a positive effect of the resistance on resistant populations. This research provides a novel genetic resource for onion thrips and a comprehensive molecular examination of resistant populations to spinosad. Those resources are important for future studies concerning thrips and resistance in insect pests regarding agriculture.

## 1. Introduction

In the past two decades, the onion thrip, *Thrips tabaci* Lindeman (Thysanoptera: Thripidae) has become a global pest of increasing concern in many crops, especially Allium species. The onion thrip has become a major pest, mainly due to its ability to develop resistance to insecticides and transmit plant pathogens along with its high reproduction rate [1]. The use of insecticides is currently the most common method to control thrips in agricultural crops; however, this is only partially effective due to the development of resistance among field populations [2]. Thrip populations have developed resistance to most of the insecticides used to control them [2,3,4,5,6,7,8,9]. Spinosad, an effective insecticide against thrips, is being used extensively by growers. Over the past few years, populations of onion thrips resistant to spinosad have been found in several location in Israel [2].

Spinosad is a macrocyclic lactone mixture (made of a spinosyn A and spinosyn D) produced from the actinomycete Saccharopolyspora spinosad [10,11,12]. Spinosad toxicity is associated with widespread neuronal excitation [13], and its target sites are nicotinic acetylcholine receptors (nAChRs) [14,15,16] and, to some extent, the γ-aminobutyric acid (GABA) receptor [17].

Resistance to spinosad was found in several insect species [18,19,20,21,22,23,24], including the onion thrip [2] and the western flower thrips *Frankliniella occidentalis* [25,26]. In twelve studies that examined the mechanisms of resistance to spinosad in several insect species, 67% of those studies reported target site resistance, 25% reported metabolic resistance, and 8% reported multiple mechanisms [22]. In the western flower thrips, the resistance to spinosad resulted from a point mutation (G275E) in the nAChR α6 subunit [27].

Advances in next generation sequencing technologies enabled exploration of the genetic changes associated with resistance to insecticides in many insect pests, which is important for agriculture and human health [28,29,30,31,32,33]. In this study, we performed de novo transcriptome sequencing to compare different populations of onion thrips that were resistant and susceptible to spinosad using the Illumina HiSeq2000 sequencing platform. We identified genes that were expressed differently in these populations that may be involved in the mechanisms of resistance and further studied the fitness of those populations that could be influenced by the resistance.

## 2. Materials and Methods

### 2.1. Insects Rearing and Resistance Bioassay

Onion thrip populations were collected from chive greenhouses in Israel. The highly resistant (HR) population was collected from Kmehin in 2007 and the susceptible (S) population was collected from Newe Ya’ar during the period 2011–2012. The populations were maintained in growth chambers in 24 ± 1 °C and a photoperiod of 16:8 L:D. The medium resistant (MR) population was established from the S population by exposure to increasing concentrations of spinosad until the desired level of resistance was obtained under laboratory conditions. The selection was carried out through five non-consecutive exposures to spinosad: Green bean seedlings; Phaseolus Vulgaris, were dipped in 0.1 ppm spinosad (diluted in distilled water) and then dried. Later on, the bean and 150 adult thrips we introduced into jars (500 mL volume) for 72 h. After 72 h, the surviving thrips were collected and transferred to normal rearing conditions, as described previously [34]. The samples used in this study were collected from the following life history stages: second instar nymph, pupa (8–11 days after hatching), and adult females. Insecticide resistance bioassays were performed with females using a modified Münger cell method [34].

### 2.2. RNA Extraction and Sample Preparation for Sequencing

Total RNA was isolated using Tri Reagent (Sigma–Aldrich, St. Louis, MO, USA), according to the manufacturer’s instructions. One hundred fifty females (2–8 days after emergence) or 400 2nd instar nymphs (3–6 days after hatching) per sample were used. For the quantitative RT-PCR (qRT-PCR) analysis, RNA from females was used, and for the transcriptome analysis a 1:1 mix of females and nymph RNA was used. RNA quantities were measured using a NanoDrop spectrophotometer (Nanodrop Technologies Inc., Wilmington, DE, USA), and its integrity was confirmed by running a 1% agarose gel electrophoresis. The RNA for qRT-PCR was cleaned before the reaction with a Turbo DNA-free kit (Ambion^®^, Austin, TX, USA) after which cDNA was synthesized using M-MLV reverse transcriptase (Promega corporation, Madison, WI, USA) according to the manufacturer’s instructions.

### 2.3. cDNA Library Construction and Illumina Sequencing

The following process was performed by BGI (Shenzhen, China), following the protocols described previously with modifications [29]. The cDNA library was constructed using an mRNA-Seq assay for paired-end transcriptome sequencing. Poly(A) mRNA was isolated from 20 mL total RNA using Oligo (dT) magnetic beads and then was broken into short fragments (200–700 nucleotides) with the presence of fragmentation buffer at 94 °C for 5 min. These short fragments were used as templates for first-strand cDNA synthesis using random hexamer primers. The second-strand cDNA was synthesized using buffer, dNTPs, RNaseH, and DNA polymerase I. Purification of short fragments with a QiaQuick PCR Purification Kit (Qiagen, Venlo, The Netherlands) and washing with EB buffer for end separation and single nucleotide adenine addition were then performed. Finally, the short fragments were connected to sequencing adapters. Suitable fragments, as judged by agarose gel electrophoresis, were enriched with PCR amplification to prepare the sequencing library. The cDNA library was sequenced on channels of an Illumina HiSeq™ 2000 instrument for 2 gigabase in-depth, which was used to obtain more detailed and better representation about the expressed genes. We sequenced four samples, two of the S population (Sus1, Sus2) and two of the HR population (Res1, Res2).

### 2.4. De Novo Transcriptome Assembly and Functional Annotation

The clean reads were generated by removing reads with adaptor sequences, reads with 5% < unknown base reads, and low quality reads from the raw reads. Then, de novo transcriptome analysis of the clean reads was carried out using the short read assembly program “Trinity” [35]. Briefly, clean reads with specified lengths of overlap were combined to form longer contiguous sequences (contigs), and then these reads were mapped back onto the contigs. The distance and relation among these contigs could be calculated based on paired-end reads, which enabled the detection of contigs, whether from the same transcript or not, and also the calculation of the distances among these contigs. Finally, we further assembled contigs using Trinity; the contigs that could not be extended on either end were defined as unigenes (unique transcripts). All the unigenes were compared to protein databases including nr, Swiss—Prot, Kyoto Encyclopedia of Genes and Genomes (KEGG), and Cluster of Orthologous Groups of proteins (COG) using the BLASTx alignment (E value < 10^−5^). The best matches were used to determine the unigenes direction. When different databases conflicted with each other to determine sequence direction, a priority order of nr, Swiss-Prot, KEGG was followed. For unigenes that did not match in the BLASTx search, we used the “ESTScan” program [36]. When direction could be determined, we presented the unique transcripts in the 5′-3′ end, otherwise they were oriented by the assembly software. For the nr annotations, the “BLAST2GO” program was also used for obtaining Gene Ontology (GO) annotations for unigenes [37]. Later, “WEGO” software was used to predict GO function classifications for the unigenes [38].

### 2.5. Reverse Transcription-PCR and Quantitative Real Time PCR (qRT-PCR)

To validate the expression levels of candidate differentially expressed genes (DEGs), a qRT-PCR analysis was performed. Total RNA was reverse transcribed with oligo(dT) and random hexamer mix (3:1, respectively) using M-MLV reverse transcriptase according to the manufacturer’s instruction (Promega, Madison, WI, USA). Blue qPCR SYBER green mix (Thermo fisher scientific, Waltham, MA, USA) and the RotorGene 3000 qRT-PCR machine (Corbett Research, Sydney, Australia) were used according to the manufacturer’s instructions. The list of primers used for these reactions is presented in Table 1. Each reaction was performed with the following steps: 95 °C for 15 min followed by 40 cycles of 95 °C for 15 s, 58 °C for 30 s’, and 72 °C for 30 s’. The Tubulin gene for which the sequences was obtained from the de novo assembly results was used as an internal reference gene (see Table 1 for primers).

To ensure the validity of the data, each target gene was amplified at least twice, and four samples per population and triplicates for each sample were used. The data was analyzed with independent t test (α = 0.05) and used a triplicate average expression level for each sample.

### 2.6. Fecundity Experiments

Two rounds of experiments were performed, and in each round eight females of the tested populations were used. The females were separated at the pupal stage to ensure that they were virgins. Those female were transferred to a 500 mL jar and two males were added in addition to a bean pod for oviposition. Every three days, the bean pod was replaced with a new one and the old pod was transferred to another 500 mL jar. The pods were changed as long as the females lived but not more than 6 times. The emerging larvae from the laid eggs were counted when they reached the 2nd instar. When the females emerged, their fecundity was determined, as described above, using a stereo microscope, then they were collected for the experiment. Results (number of larvae per female) were analyzed using two way ANOVA followed by the Tukey HSD test (α = 0.05).

## 3. Results

### 3.1. Transcriptome Sequencing, Assembly and Annotation

**Figure 1:**
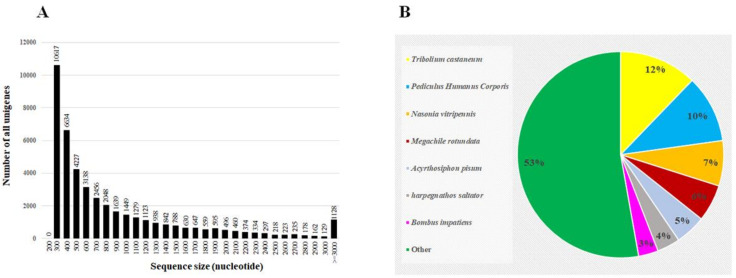
Length distribution of all the assembled unigenes generated from the sequenced samples (**A**), and homologies of the unigenes to known insect sequences in GenBank (**B**).

Three populations were used in this study, and their levels of resistance to spinosad were identified, as summarized in Table 2. Four RNA samples were subjected to RNAseq analyses: two samples from the S strain (named Sus1 and Sus2) and two from the HR strain (named Res1 and Res2). The GC content (%) was 54.91–56.29 and the Q20 (%) was 97.06 to 97.18. Total number of bp reads, assembled unigenes, and the mean length of unigenes in each sample are given in Table 3; 76,237–79,934 contigs per sample with mean lengths of 349–363 bp were assembled. The unigenes length distribution is presented in Figure 1. The data generated was submitted to the National Center for Biotechnology Information (NCBI) Short Read Archive (SRA) with the accession number SRX690526.

Altogether, 43,843 unigenes were assembled from the different samples that were subject to RNAseq and were run against the different online databases. Out of the assembled unigenes, 25,552 (58.26%) were functionally annotated, and the highest matches were to the red flour beetle Tribolium castaneum (12.2%) and to other well-known arthropods (Figure 1).

**Figure 2:**
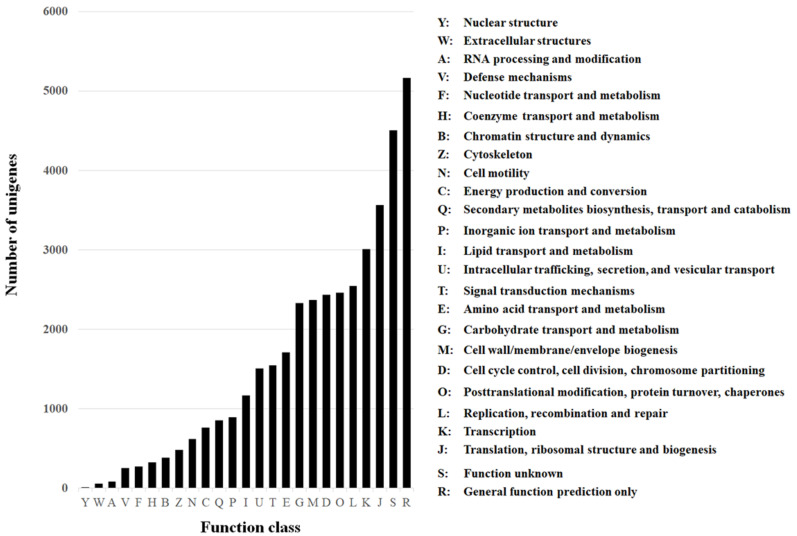
Cluster of Orthologous Groups (COG) of the unigenes obtained from sequencing the resistant and susceptible samples used in this study.

Of the annotated genes, 24,691 (96.63%) had a significant match to the National Center for Biotechnology Information (NCBI) non-redundant protein (nr) database, 10,642 (41.65%) had a significant match to the NCBI non-redundant nucleotide sequence (nt) database, 22,067 (86.36%) had a match to the Swissprot database, 19,977 (78.18%) to the Kyoto Encyclopedia of Genes and Genomes (KEGG), 11,971 (46.85%) to the Cluster of Orthologous Groups of proteins (COG) (Figure 2), and 11,943 (46.74%) to the Gene Ontology (GO).

**Figure 3:**
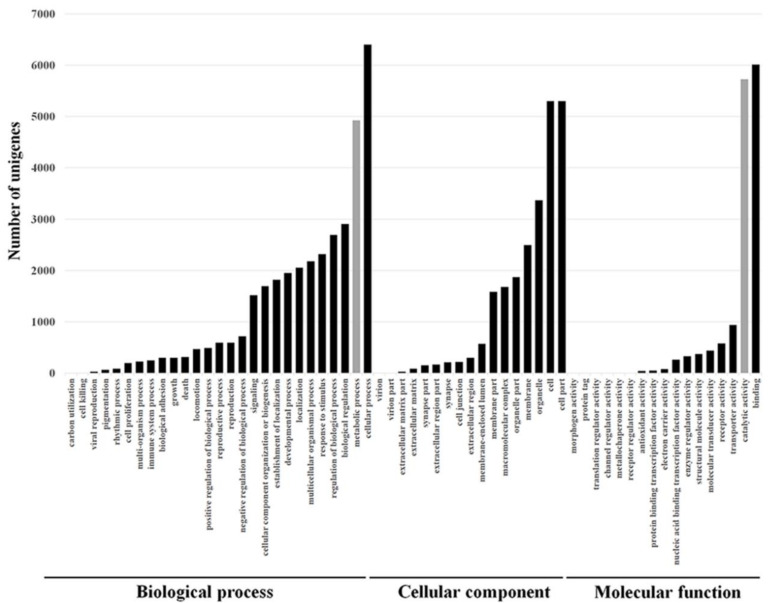
Gene Ontology (GO) classification of assembled unigenes from *thrips tabaci* transcriptome into biological process, cellular component, and molecular unction presented as absolute number of unigenes in each subcategory. The categories were obtained using Blast2GO website.

For the GO, the unigenes were divided into three ontologies: 51.8% of the unigenes belonged to the biological process category, 26.4% to the cellular component category, and 21.8% to the molecular function category (Figure 3). Among the different GO categories, 5724 and 4926 unigenes that matched the catalytic activity (GO:0003824) and metabolic processes (GO:0008152) ontologies, respectively, were identified (Figure 3, gray columns). These groups are of special interest because metabolic detoxification enzymes are expected to belong to these groups, and those enzymes are involved in resistance to insecticides. The KEGG annotation database matched 19,977 unigenes in 258 predicted pathways. The pathway that involved the highest number of unigenes was “Metabolic pathways” (ko01100) with 2464 (12.3%) unigenes. Another interesting pathway that was represented by 132 unigenes (0.66%) was “Metabolism of xenobiotics by cytochrome P450” (ko00980), and these are expected to be involved in resistance. A large numbers of genes belonging to known families of detoxification enzymes including 260 cytochrome P450 (CYP450), 863 esterases (among them 128 carboxylesterase), and nine glutathione s-trasnsferase (GST) were identified.

### 3.2. DEGs and Resistance Related Genes

**Figure 4:**
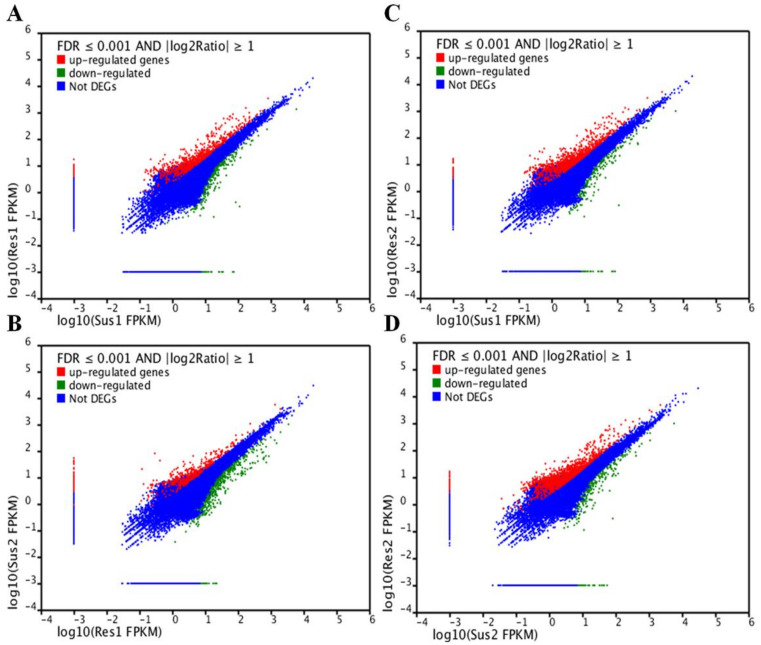
Differently Expressed Genes (DEGs) in resistance compared to susceptible strains shown in a graphic representation for each comparison: Sus1 vs. Res1 (**A**), Sus1 vs. Res2 (**B**), Res1 vs. Sus2 (**C**), Sus2 vs. Res2 (**D**). In the tested onion thrip populations, genes were considered DEG if False Discovery Rate (FDR) ≤ 0.001 and log2 (sample1 expression/sample2 expression) ≥ 1. Unigenes shown in red are significantly up regulated and those shown in green are significantly down regulated.

In order to identify DEGs, the expression level of all the unigenes in the two susceptible samples—Sus1, Sus2—were compared with the two resistance samples—Res1, Res2. In total, four comparisons were carried out (Sus1 vs. Res1, Sus1 vs. Res2, Sus2 vs. Res1, Sus2 vs. Res2). In addition, two comparisons within the susceptible or resistance samples (Sus1 vs. Sus2, Res1 vs. Res2) were carried out as well. These comparisons enabled us to verify that a DEGs with differential expression in the resistance compared to the susceptible population was not differentially expressed within the compared populations. A unigene was considered as a DEG if its normalized expression level (fragments per kb per million fragments (FPKM)) ratio in one sample compared to the other was at least double (Log2[FPKM1/FPKM2] ≥ 1) and its corrected *p* value (False Discovery Rate (FDR)) was FDR ≤ 0.001. Based on those criteria, more than 1800 DEGs were identified (Figure 4).

An enrichment analysis for identifying clusters of GO terms that were enriched with DEGs was carried out. The GO terms “Structural constituent of Cuticle” (GO:0042302) and the term “Metabolic Process” (GO:0008152) were significantly enriched with DEGs. For the first term, 3.6% of the clustered unigenes were DEGs with *p* = 1.65 × 10^−7^ and, for the second term, 69.4% of the clustered unigenes were DEGs with *p* = 1.12 × 10^−3^.

In order to further focus on specific DEGs that may be involve in the resistance mechanism, DEGs that encode enzymes belonging to known detoxification mechanisms were searched for. The search focused on genes encoding for cytochrome p450 monooxegenases (Cyp450), Glutathion S transferases (GSTs), and esterases. These three groups are known as the main xenobiotics detoxification enzymes groups in insects and other organisms [39]. Additional groups of genes that were of high interest were genes encoding for cuticular proteins due to their potential role in reduced penetration resistance mechanisms (resistance due to lower toxin penetration rates through the cuticle), and ion channels due to their important role in resistance as target site mutations mechanisms against many insecticides [40].

Except for the DEGs that were of interest due to their potential role in resistance mechanisms, DEGs that were differentially expressed at exceptionally different folds between the compared susceptible and resistant populations (│Log2[FPKM1/FPKM2]│ ≥ 8) were also recorded (Table 4).

### 3.3. Verifying Expression Levels of Candidate Gene Using qRT-PCR

**Figure 5:**
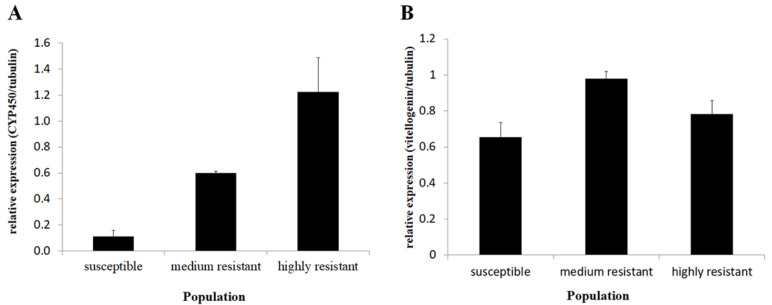
(**A**) Cytochrome P450 (CL844) relative expression levels in the three lab populations. The quantification was conducted using qRT-PCR (ANOVA, *n* = 4, df = 2, *p* = 0.002). (**B**) Relative expression levels of vitellogenin (CL1604) in the three thrip populations. Quantification was done using qRT-PCR (ANOVA, *n* = 4, df = 2, *p* = 0.022).

The expression levels of the selected candidate genes that were highly expressed and included CYP450 (CL844) and Vitellogenin (CL1604) were measured with qRT-PCR in order to verify the expression differences obtained in the RNAseq analysis. Similar trends to the transcriptome results were obtained, suggesting valid results in both experiments (Figure 5). The CYP450 average expression level differed between the tested populations (ANOVA, *n* = 4 df = 2, *p* = 0.002). The expression level in the HR population was 10.94 fold higher than in the S population (Tukey HSD, *p* = 0.002). The expression level in the MR population (selected) was intermediate and did not differ from the HR or S populations (Tukey HSD, *p* > 0.05).

The vitellogenin expression level differed between the populations (ANOVA, df = 2, *p* = 0.022).

The average expression level was 1.2 fold higher in the HR population compared to the S population, but they were not significantly different (Tukey HSD, *p* > 0.05). The expression level in the MR population was 1.49 fold higher than in the S population (Tukey HSD, *p* = 0.018), but did not differ significantly from the HR population (Tukey HSD, 0 *p* > 0.05).

### 3.4. Fecundity of Resistant and Susceptible Populations to Spinosad

Vitellogenin is the precursor for vittelin, the egg yolk protein, which has an important role in egg production, development, and reproduction [42]. Because significant differences in its expression were identified in the previous experiments, and because resistance to insecticides has fitness cost, as has been previously shown in many insect orders and types of resistance [43], fecundity experiments were carried out to examine whether the expression levels of this gene are correlated with fecundity differences and the different levels of resistance to spinosad.

**Figure 6:**
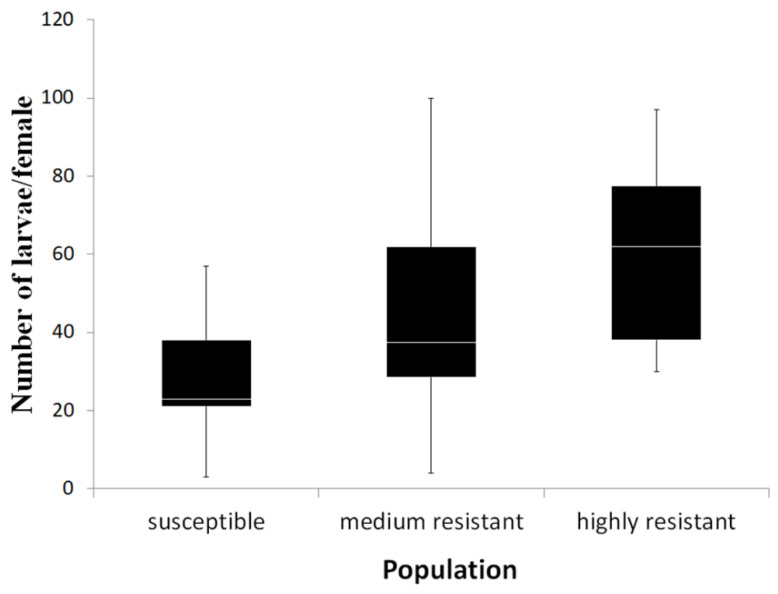
Fecundity presented as the number of larvae (2nd instar) per female in the three thrip populations used (ANOVA, *n* ≤ 13, df = 2, *p* = 0.006).

In order to estimate the fecundity in the different populations, the number of offsprings (second larvae) per female was counted. The fecundity differed significantly between the populations (ANOVA, df = 2, *n* ≤ 13, *p* = 0.006) (Figure 6). The fecundity of HR females was 60.5 ± 6.0 (mean ± SE), and it was significantly higher than the S females—28.7 ± 4.5 (Tukey HSD, *p* = 0.002). The fecundity of MR females was intermediate—45.9 ± 7.0—and did not differ from females of the other populations (Tukey HSD, *p* > 0.05).

## 4. Discussion

Insecticide resistance is a major problem limiting pest control worldwide. So far, there are at least 82 reports of onion thrip insecticide resistant populations globally [44]. In this study, we performed a transcriptomic comparison of spinosad resistant and susceptible onion thrip populations. The 25,552 annotated unigenes is a valuable resource that can be used in future studies dealing with thrip species in general, and onion thrips in particular, including studies concerning mechanisms involved in spinosad resistance in this and other insect species.

The HR population used in this study was highly resistant compared to other reported onion thrip and western flower thrip resistant populations [2,25]. The MR population had a similar genetic background as the S population. Thus, it was used to refute the possibility that differences between HR and S populations are due to genetic differences.

The de novo transcriptome sequencing and analysis generated a large amount of data. The quality parameters of the sequencing and the assembly processes, such as the Q20 values and the assembled contigs and unigenes numbers and their length distribution, indicated that these processes were carried out at a sufficient level in order to represent reliably the transcriptome under the studied conditions. Following transcriptomics, it was possible to functionally annotate large amount of unigenes, similar to other non-model organisms transcriptomic studies [28,29,33]. High percentage of unannotated unigenes could be due to insufficient sequence availability in public databases for phylogenetically closely related thrip species. In addition, because the significance of the annotation comparison depends in part on the length of the query sequences, short reads obtained from sequencing would rarely be matched to known genes [45,46]. Another parameter that can affect the assembly and therefor the percentage of annotated sequence is the sequencing depth or coverage. The coverage is the average number of times that each nucleotide is sequenced [47]. In this work, we generated two Giga base pairs of clean data per sample, which provided an adequate annotation percent. Among the assembled unigenes, we found homology to genes from a wide range of insect species and orders including sequences from onion thrips and western flower thrips, for which a whole genome sequence is now underway; however, the data are not yet released for data queries and comparisons.

The functional annotation of the whole transcriptome can provide a general gene expression profile signature for the organism [23], much like the expression classification profiles obtained from the Cluster of Orthologous Groups (COG) database (Figure 2), or the Gene Ontology classification (Figure 3). The main strength of these databases is that they group genes under the same biological or molecular functions. Thus, the genes of interest can be fished out after analysis, such as was the case in this project in which resistance-related genes like “metabolism of xenobiotics by cytochrome P450” were identified. Here, a large number of genes belonging to known families of detoxification enzymes including CYP450s, esterases, and GSTs were identified [39]. In addition, we identified several significantly-regulated genes that encode insecticide target proteins such as voltage gated chloride channels, GABA receptors, and nAChRs. The later is especially interesting because it is the main target protein of spinosad [13,15,16]. Future studies should address the question of whether the mutations associated with spinosad resistance in several insect species also exists in onion thrips [22,27,48,49].

In order to focus on DEGs that could be possibly involved in the resistance mechanisms, certain criteria to filter the 1800 DEGs were used. The criteria were based on two main aspects: the levels of gene expression differences between the tested populations and the known role in resistance mechanisms. In order to meet these criteria, several gene groups among the most differently expressed genes were searched. These groups included detoxification enzymes, ion channels, and cuticle proteins. Three CYP450 encoding genes were among the detoxification enzymes that were exceptionally up-regulated (Table 4 and Table 5) [50]. CYP450s belong to an important metabolic system involved in the catabolism and anabolism of xenobiotics and endogenous compounds. This is a large group of proteins founds in many organisms, including insects, and is the largest group of proteins associated with insecticide resistance in insects [51,52,53]. The three main differentially-expressed CYP450s that we identified here belong to family 4, which is known to be associated with insecticide resistance in several cases and has been shown to be up-regulated in insects that are resistant to several chemical groups of insecticides [39]. The polyphagous nature of the onion thrip makes it prone to evolving and using detoxification mechanisms such as CYP450, because these systems are widely used by polyphagous insects when encountering different plant secondary metabolites during feeding [39]. The expression level of CYP450 (CL844) was validated using qRT-PCR, and similar trends were observed, including elevated expression (though not significant) in the MR population. These findings suggest that this specific CYP450 is associated with spinosad resistance possibly through metabolic detoxification, although studies have shown that the main mechanism associated with resistance to spinosad, especially in the western flower thrip, is mainly due to a target site mutations [22,27,48,49]. Other studies have shown that metabolic resistance is also an important mechanism in spinosad resistance; for example, in the cotton bollworm Helicoverpa armigera [22,54,55,56]. The involvement of several resistance mechanisms in conferring resistance to the same chemical compound is not rare, and many previous cases have been documented [57,58,59].

An additional DEG group of interest includes the genes that encode cuticle proteins. Changes in the cuticular structure or chemical composition can reduce the susceptibility of insects to insecticide by reducing the insecticide penetration rate. A thicker cuticle or different hydrocarbon cuticle composition were discovered in pyrethroid resistant strains of Triatoma infestans [59], Anopheles funestus [60], and H. armigera [56]. In our current study, 59 differently regulated cuticular unigenes were identified. Among these, two were exceptionally up-regulated in the HR population (Table 4 and Table 5).

Three more groups of genes have met the selection criteria, but their possible role in spinosad resistance was not clear. The first is the voltage gated chloride channel, for which two unigenes were up-regulated in the HR population compared to the S population (Table 4 and Table 5). The voltage gated chloride channel is known to be a target site, among several other sites, for pyrethroids but not for spinosad [61,62,63]. Another interesting DEG was a cysteine protease. This gene was up-regulated in the susceptible population. Cysteine proteases play a wide range of roles in insects including functions in molting [64], resistance against plant defensive proteins [65], immune responses [66], digestion [67], and embryogenesis (vitellogenin processing) [68,69,70,71,72,73]. An additional interesting DEG that we identified is vitellogenin (Vg). Vg is the precursor of the egg yolk protein vittelin, and it has a fundamental role in oocyte growth and maturation during vitellogenesis [42,74]. In the transcriptome results generated here, Vg was up-regulated in the HR population, although the qRT-PCR validation was not significant. On the other hand, there was a significant up-regulation in the MR population compared to the S population. Because Vg and cysteine protease were identified as differentially regulated, differences in the fecundity among the tested populations was investigated, as those genes have roles in reproduction and egg production. The results showed that the HR population was more fecund compared with the S population. The MR population showed intermediate fecundity between the S and HR populations, indicating that the gene expression results are partially correlated with the fecundity experiment, suggesting a biological relevance of the obtained results. These results were somehow surprising because, in many cases, resistance has been associated with fitness costs such as a decrease in fecundity or body size and developmental time [75]. However, in some cases, accumulating evidence suggested increase in fitness parameters, such as fecundity and fertility, among insecticide resistance populations [75,76]. In the western flower thrip for example, a spinosad resistant population has been shown to be more fecund than a susceptible one [75]. In the current study, we measured only one parameter of fitness, thus an overall conclusion regarding other fitness cost parameters and overall fitness cannot be concluded. The fecundity differences that were identified here are especially interesting in light of the expression of the cysteine protease and Vg genes. These expression and fecundity differences were not linearly correlated, but showed a trend and association that could be addressed in future studies.

## Figures and Tables

**Table 1:** Primer sequences and characteristics of the primers used for qRT-PCR.

Name of Primers	Target Gene	Sequence (5′ > 3′)	Product Size (bp)	Tm
CL585C5-F	Tubulin (CL585)	TTCCACTGCTGTTGTTGAGC	98	58
CL585C5-R		AGATGGCCTCATTGTCAACC		
CL844- F	CYP450 (CL844)	CTGGGACTTGTTGTGGACCT	175	58
CL844-R		GGACTTTTCGGTGTGCTTGT		
CL1604C5-F	Vitellogenin (CL1604)	TTTTTGATTGTGACCGACCA	62	58
CL1604C5-R		CAAAAGTCCAGCACCCAGTT		

**Table 2:** Onion thrip populations used in this study and their susceptibility to spinosad.

Population Name	LC_50_ (Range) ppm ^a^	Slope ^a^	RR_50_^b^
S	0.6 (0.5–0.7)	1.6 ± 0.2	1.0
MR (selected)	117.2 (92.2–167.6)	0.1 ± 0.0	212.7
HR	23258.0 (15660.1–55825.2)	0.001	42,210.6

^a^ The values were calculated using Probit model. ^b^ The values were calculated by dividing the LC_50_ by the S population LC_50_.

**Table 3:** Summary of Illumina transcriptome sequencing and assembly for the onion thrips.

Sample	Sus1	Sus2	Res1	Res2
Total clean Reads	26,761,394	26,187,172	27,162,192	26,163,866
Unigenes number	47,927	44,380	48,605	47,150
Unigenes mean length	581	581	575	609

**Table 4:** The most differentially expressed genes (DEGs) in the HR (resistant) vs the S (susceptible) populations.

Gene ID	Potential Function	Expression Ratio ^a^	FDR ^b^
Unigene22894	Cytochrome P450	5.67	FDR ≤ 2.310^−24^
CL844	Cytochrome P450	5.07	FDR ≤ 2.40 × 10^−26^
Unigene20207	Cytochrome P450	3.25	FDR ≤ 2.26 × 10^−14^
CL490	voltage gated chloride channel	11.88	FDR ≤ 2.29 × 10^−38^
Unigene9317	voltage gated chloride channel	11.17	FDR ≤ 5.97 × 10^−75^
CL353	cuticle protein	8.35	FDR ≤ 1.38 × 10^−30^
CL2493	cuticle protein	9.8	FDR ≤ 5.58 × 10^−164^
CL2817	cysteine protease	0.0025	FDR ≤ 2.66 × 10^−100^
CL1604	Vitellogenin-5	17.06	FDR ≤ 15.15 × 10^−5^

^a^ The average expression ratio (Expression _resistant_/Expression _susceptible_) in the four comparisons made (in the two susceptible samples versus the two resistant samples). ^b^ The maximal FDR value in the four comparisons made. The FDR level was calculated using the significance of digital gene expression profiles [41].

**Table 5:** The functional annotation information regarding the DEGs from Table 4.

Gene ID	Annotated Gene	database	Accession Number	E Value	Gene Ontology (GO)
Unigene 22894	cytochrome P450 4g15-like (*Bombus terrestris*)	Nr	XP_003399611.1	2 × 10^−109^	GO:0044464, cell part
CL844	cytochrome P450 (*Bemisia tabaci*)	Nr	AEK21806.1	1 × 10^−126^	GO:0046872,metal ion binding/GO:0016491, oxidoreductase activity/GO:0016020, membrane
Unigene 20207	cytochrome P450 enzyme, CYP4C39 enzyme (*Carcinus maenas*)	Nr	JC8026	5 × 10^−56^	GO:0044464, cell part
CL490	voltage gated chloride channel domain-containing protein (*Toxoplasma gondii*)	Nr	XP_002368679.1	2 × 10^−6^	−
Unigene 9317	voltage gated chloride channel domain-containing protein (*Toxoplasma gondii*)	Nr	XP_002368679.1	9 × 10^−6^	−
CL353	cuticle protein (*Nasonia vitripennis*)	Nr	XP_001606311.1	7 × 10^−12^	−
CL2493	cuticle protein 16.5 Isoform A (*Locusta migratoria*)	Swiss-prot	P83992	3 × 10^−20^	GO:0042302, Molecular Function
CL2817	cysteine protease CP19 precursor (*Frankliniella occidentalis*)	Nr	AAL02223.1	6 × 10^−67^	GO:0016787, hydrolase activity
CL1604	Vitellogenin-5 (*Camponotus floridanus*)	Nr	ADZ13687.1	2 × 10^−8^	−

## Data Availability

The raw data generated in this study was submitted to the National Center for Biotechnology Information (NCBI) Short Read Archive (SRA) with the accession number SRX690526.
